# Expectant Fathers’ Mental Health History Predicts Actual Depressive Symptomatology in Pregnant Women

**DOI:** 10.1192/j.eurpsy.2023.1358

**Published:** 2023-07-19

**Authors:** M. Bottaro, I. G. Ylmaz-Karaman, L. Orsolini, S. Pompili, S. Bellagamba, S. Pacini Biagiotti, U. Volpe

**Affiliations:** 1 University Psychiatric Clinic, Department of Clinical Neuroscience/DIMSC, Università Politecnica delle Marche, Ancona, Italy; 2 Department of Psychiatry, Faculty of Medicine, Eskişehir Osmangazi University, Eskişehir, Türkiye

## Abstract

**Introduction:**

Peripartum period is a risky period for mental ill health among women. Biologically endocrinal changes, pregnancy complications, and lack of sleep due to childcare may increase psychopathology. From a social perspective, there is a role translation from women into mother, which is highly demanding. Moreover, the psychological approach underlines interpersonal relationships during the peripartum period. Even though the clinical focus is on postpartum depression, research shows its roots in pregnancy depression.

**Objectives:**

The present study aims to detect predisposing factors to pregnancy depression.

**Methods:**

One hundred-six pregnant women admitted to Salesi Pediatric Hospital of Ancona, participated in the study between April 2021- February 2022. Participants completed the sociodemographic form and Edinburgh Postpartum Depression Scale (EPDS). EPDS scores higher than or equal to 9 are considered psychometric depression.

**Results:**

The mean age of participants were 33.30 ± 4.64. Most were Italian (97.2%) and cohabiting/married (97.2%). Almost half of the participants were university graduates (50.9%). 84% were employed. The pregnant women were predominantly in the third trimester (71.7%). 58.5% had no children before. No participants were using alcohol or drugs. Pregnancy depression was 13.2% prevalent (See Table 1). Table 2 summarizes binary logistic regression analysis: Higher age, gestational comorbidity, and pregnant women’s and their partner’s psychiatric disorder history predicted depressive symptoms above the threshold.

**Image:**

**Figure:**
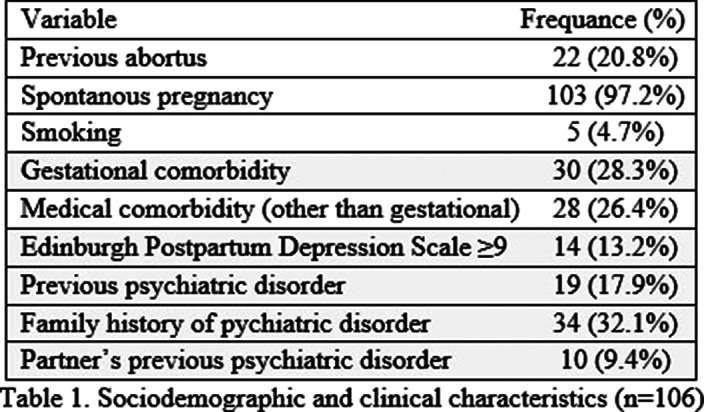

**Image 2:**

**Figure:**
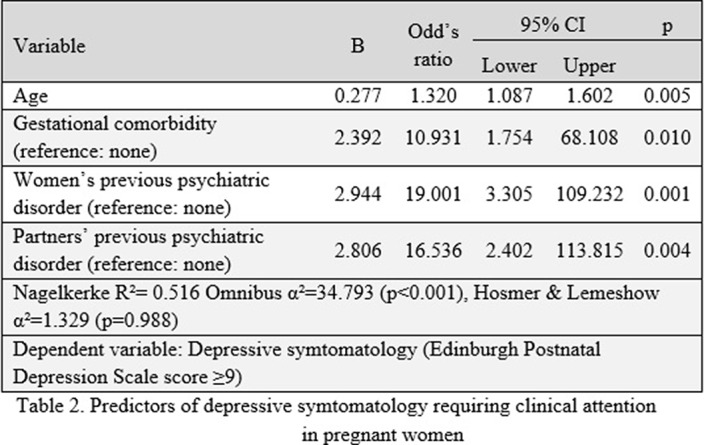

**Conclusions:**

Our study reveals well-known risk factors for pregnancy depression and a new finding: expectant fathers’ mental health history predicts actual depressive symptomatology in pregnant women. Fathers should be included in perinatal mental health care. Prevention programs targeting peripartum depression should cover fathers’ mental health.

**Disclosure of Interest:**

None Declared

